# Paroxysmal Permeability Disorders: Development of a Microfluidic Device to Assess Endothelial Barrier Function

**DOI:** 10.3389/fmed.2019.00089

**Published:** 2019-04-24

**Authors:** Maddalena Alessandra Wu, Daria Tsvirkun, Lionel Bureau, Isabelle Boccon-Gibod, Mehdi Inglebert, Alain Duperray, Laurence Bouillet, Chaouqi Misbah, Marco Cicardi

**Affiliations:** ^1^Department of Biomedical and Clinical Sciences, ASST Fatebenefratelli Sacco, Luigi Sacco Hospital, University of Milan, Milan, Italy; ^2^Univ. Grenoble Alpes, LIPHY, Grenoble, France; ^3^CNRS, LIPHY, Grenoble, France; ^4^Belozersky Institute of Physico-chemical Biology, Lomonosov Moscow State University, Moscow, Russia; ^5^Department of Internal Medicine, Centre Hospitalier Universitaire de Grenoble, Grenoble, France; ^6^Univ. Grenoble Alpes, IAB, Grenoble, France; ^7^INSERM, IAB, Grenoble, France; ^8^IRCCS-Istituti Clinici Scientifici Maugeri, University of Milan, Milan, Italy

**Keywords:** endothelial permeability, endothelial function, Paroxysmal Permeability Disorders, microfluidic device, microchip, shear stress, angioedema, idiopathic systemic capillary leak syndrome

## Abstract

**Background:** Paroxysmal Permeability Disorders (PPDs) are pathological conditions caused by periodic short lasting increase of endothelial permeability, in the absence of inflammatory, degenerative, ischemic vascular injury. PPDs include primary angioedema, idiopathic systemic capillary leak syndrome and some rare forms of localized retroperitoneal-mediastinal edema.

**Aim:** to validate a microfluidic device to study endothelial permeability in flow conditions.

**Materials and Methods:** we designed a microchannel network (the smallest channel is 30μm square section). Human Umbilical Vein Endothelial Cells (HUVECs) were cultured under constant shear stress in the networks. Endothelial permeability assessment was based on interaction of biotinylated fibronectin used as a matrix for HUVECs and FITC-conjugated avidin. The increase in endothelial permeability was identified as changes in fluorescence intensity detected by confocal fluorescent microscopy.

**Results:** The microchannels were constantly perfused with a steady flow of culture medium, ensuring a physiologically relevant level of shear stress at the wall of ~0.2 Pa. Our preliminary results demonstrated that circulation of culture medium or plasma from healthy volunteers was associated with low fluorescence of fibronectin matrix. When bradykinin diluted in culture medium was perfused, an increase in average fluorescence was detected.

**Conclusion:** Our microvasculature model is suitable to study endothelial functions in physiological flow conditions and in the presence of factors like bradykinin known as mediator of several PPDs. Therefore, it can be a promising tool to better understand the mechanisms underlying disorders of endothelial permeability.

## Background

The vascular endothelium is considered as a complex organ, which is responsible for the dynamic control of vessel functions, such as the transport of fluids and proteins from the intra- to the extravascular space (and vice versa), the modulation of the immune response, the regulation of the balance between procoagulant and anticoagulant factors, nutrients' trafficking, angiogenesis and the orchestration of organ development (1). The endothelium is continuously exposed to shear stress and changes in pressure, including rhythmic fluctuations due to heart beating. Endothelial cells (ECs) are physiologically separated by an intercellular space of 6–8 nm. ECs' membrane proteins allow tightly controlled and regulated passage of water, gases, electrolytes and small molecules through this intercellular space. Fluid exchange between intravascular and extravascular space occurs via diffusion and filtration. Proteins cross the vascular wall via either paracellular and transcellular pathways. The first one is controlled by the dynamic opening and closing of interendothelial junctions (2), the latter includes vesicular transport systems, fenestrae, and biochemical transporters (3).

The sieving properties and permeability of the endothelium depend on the function of specialized selective junctional regions which link ECs one to another. These junction regions are organized mainly by two types of contacts with different functions: adherens junctions (AJs) initiate cell-to-cell contacts and promote their maturation and maintenance; tight junctions (TJs) regulate the passage of ions and solutes through the paracellular route (4–6). ECs express cell-type-specific transmembrane adhesion proteins such as VE-cadherin (also known as CD144) as a core protein of AJs and claudin-5 at TJs (7).

VE-cadherin is expressed in essentially all types of vessels. Different mediators including histamine, tumor necrosis factor, platelet activating factor, and vascular endothelial growth factor as well as changes in fluid shear stress are able to induce phosphorylation of VE-cadherin, β-catenin, and p120. This phosphorylation results in an increased permeability of the endothelial monolayer, changes in flow sensing, and vascular remodeling (2–5, 8–10). VE-cadherin may also be phosphorylated through inhibition of associated phosphatases, e.g., the VE-PTP phosphatase, which is endothelial-specific (11). Other pathways that may modify endothelial permeability are VE-cadherin cleavage and regulation of its expression (12).

Phenotypic heterogeneity is a key feature of the vascular endothelium, which displays different morphology, behavior, biosynthetic repertoire according to different sites (13–15).

Over the past decade there has been an explosion of interest in the thin (~500 nm), gel-like endothelial glycocalyx layer that coats the luminal surface of blood vessels and participates in the regulation of a wide range of vascular functions (16). It is currently known that its main functions include modulation of vessel permeability to water and macromolecules; mechanotransduction of fluid shear stress and pressure to the endothelial cytoskeleton and regulation of shear stress-mediated NO production; regulation of red and white blood cells' adhesion as well as modulation of the inflammatory response via binding of inflammatory cytokines (16). Alterations and shedding of the glycocalyx can contribute to the pathophysiology of very severe conditions such as septic or hemorrhagic shock (17, 18).

Since ECs are exposed to signals from both surrounding tissues and flowing blood, their regulatory pathways are numerous and cross interacting. It is known that several soluble mediators can influence ECs via many receptors, activating a number of intracellular pathways, which influence each other and result in multiple feedback mechanisms, able to modify endothelial permeability either directly or indirectly, rapidly or through long-term processes (19, 20). Moreover, endothelial cells' behavior might be influenced not only by hemodynamic forces but also by factors such as changes in blood oxygenation, temperature and pH. There is a huge variety of mediators, receptors, junctions and pathways, which are constantly exposed to hemodynamic forces and, as the musical instruments of an orchestra, interact one with another, each one with its specific role and timing, in order to generate a complex harmony, namely the fine modulation of endothelial permeability.

## Paroxysmal Permeability Disorders

Over the decades the term “endothelial dysfunction” has been applied to a huge variety of conditions associated with alterations of the endothelial morphology and functions.

For instance, thickening of the intima, proliferation of smooth muscle cells, formation of the fibrous plaques, changes of the vasa vasorum, loss of endothelium-derived nitric oxide, alterations of the endothelial glycocalyx, hyper-adhesiveness of the vascular lining toward platelets especially in complex hemodynamic shear stress regions are the processes lying at the basis of atherosclerosis, which is responsible for coronary, cerebral, peripheral artery and aortic diseases (21, 22).

The term “endothelial dysfunction” has been also used with reference to the loss of the ability of the endothelium to regulate vascular resistance. This alteration might imply chronic structural changes of the endothelial cell barrier as it happens in pulmonary hypertension (23). However, in some conditions the alterations of the endothelial morphology and functions are transient followed by a restoration almost to normal after the resolution of the acute phase. Among the latter conditions is for instance sepsis, which is characterized by increased leukocyte adhesion and trafficking, altered vasomotor tone, loss of endothelial barrier function, shifts in hemostatic balance and programmed cell death, even though endothelial structural changes can also occur—nuclear vacuolization, cytoplasmic swelling and fragmentation, ECs detachment (24).

It is possible to distinguish a variety of diseases which are due to recurrent alterations of endothelial permeability, with no inflammatory, degenerative, ischemic vascular injury and complete *restitutio ad integrum* after each episode. For these cases we would like to propose a sort of a new nosological entity, namely the Paroxysmal Permeability Disorders (PPDs) in the effort of grouping conditions that are due to periodic dysfunction of endothelial permeability and probably share some common pathophysiological mechanisms, although they are characterized by different clinical pictures and differ in therapeutic approaches (Table 1).

**Table 1:** Paroxysmal Permeability Disorders: features for inclusion/exclusion together with currently identifiable clinical phenotypes.

**PAROXYSMAL PERMEABILITY DISORDERS**
**CRITERIA**
**Features for inclusion** - Recurrent self-limiting local or systemic interstitial edema - For non-lethal episodes, complete healing max 1 week, mostly 1–3 days **Features for exclusion** - Any underlying clinical condition causing increase in endothelial permeability (infections, systemic inflammation, allergy, malignancy, injury, autoimmune disease etc.) - Local signs of inflammation, ischemia-necrosis, tissue degeneration
**CLINICAL PHENOTYPES**
**Primary Angioedema** - Idiopathic histaminergic angioedema - Hereditary/acquired angioedema due to C1 inhibitor deficiency - Hereditary angioedema with normal C1 inhibitor - Idiopathic non-histaminergic angioedema **Idiopathic Systemic Capillary Leak SyndromeYet poorly defined forms of periodic edema** - Recurrent retroperitoneal edema - Recurrent female periodic edema of unknown origin - Recurrent edema in patients with hypereosinophilia (Gleich's syndrome)

Among such diseases we include clinical conditions which can be rapidly lethal, due to a localized process (e.g., laryngeal edema in primary angioedema) or to a systemic derangement (e.g., hypovolemic shock due to massive plasma and protein extravasation in idiopathic systemic capillary leak syndrome). PPDs also include some yet poorly defined forms of periodic edema. Scanty data are available about the pathogenesis of some of these conditions, possible triggers and means to correctly treat them, avoiding acute and long-term complications.

### Primary Angioedema

Hereditary angioedema due to C1 inhibitor deficiency or dysfunction (C1-INH-HAE) is a rare autosomal dominant disorder (prevalence around 1:50,000 people in the general population) characterized by localized, non-pitting edema of the skin and submucosal tissues of the upper respiratory and gastrointestinal tracts, without significant wheals or pruritus, due to a temporary increase in vascular permeability. It represents the best characterized form of primary angioedema, a group of conditions where angioedema occurs in the absence of wheals and of an identified causative factor. The same group of pathological conditions include also the forms of hereditary angioedema related to Factor XII / Plasminogen /Angiopoietin 1 mutations and the forms with unknown etiology.

In these angioedema chronically recurrent symptoms cause significant personal, domestic, social, and occupational disability and exposes patients to the risk of death (due to asphyxia when the respiratory tract is involved) (25–27).

### Idiopathic Systemic Capillary Leak Syndrome

Idiopathic Systemic Capillary Leak Syndrome (ISCLS), also known as Clarkson's Disease (28), is a rare disorder (about 260 cases described) with recurrent potentially life-threatening episodes of distributive shock with hemoconcentration and hypoalbuminemia (29–32). During attacks, endothelial hyperpermeability results in leakage of water, solutes and plasma proteins (up to the size of 300kDa) into the interstitial space. The management of this condition is extremely challenging not only because of the intrinsic severity of the attacks but also because of the high risk of inducing iatrogenic damage both in the acute and post-acute phases, especially when a strategy of aggressive fluid replacement is erroneously chosen (33, 34).

### Yet Poorly Defined Forms of Periodic Edema

Among PPDs there are also conditions characterized by recurrent edema with no known trigger and/or etiology. We recently reported cases of significant isolated retroperitoneal edema (with no thickening of omentum/intestinal walls or ascites), with complete resolution after the acute crisis (35).

## Assessment of Endothelial Permeability

When investigating alterations of endothelial permeability, different kinds of strategies can be chosen. Depending on the specific clinical conditions under investigation, different parameters can be evaluated (e.g., cleaved high molecular weight kininogen in primary angioedema, hematocrit and albumin values in idiopathic systemic capillary leak syndrome). This strategy is certainly useful from the diagnosis point of view and can give some important clues to unveil some of the pathophysiological mechanisms underlying PPDs. The chase for a permeability increasing factor led Xie et al. to show that circulating angiopoietin 2 (Angpt2) and vascular endothelial growth factor (VEGF) are increased in acute but not in convalescent sera from ISCLS patients and both of them are able to induce endothelial hyperpermeability *in vitro* by disrupting endothelial adherent junctions (36). Angpt2 and VEGF cause endothelial cells' retraction without inducing cell death, with attenuation of membrane VE-cadherin and actin stress fiber formation (36). Likewise, research is ongoing to assess the role of the monoclonal component which can be found in the majority of ISCLS patients (32).

In order to investigate endothelial function, a variety of static *in vitro* models has been proposed and used in recent years and provided some relevant information to the understanding of B2 and B1 types of bradykinin receptor and gC1q receptor in the vascular leakage induced by plasma from C1 inhibitor deficient patients (37).

Microfluidic technology highly developed in physics is now widely used to create tools for cell biology (38). A variety of bioassays and investigations can be carried on in microfluidic systems where living cells can be cultured: cell migration and interaction, cancer cell invasion, drug delivery assays, wound healing, angiogenesis, thrombosis, studies of blood flow and shear stress etc. (38). The insights derived from this kind of research have potential implications to get some clues in clinical settings, both for a better understanding of some pathophysiological mechanisms (such as wound healing and cancer progression) and for searching of therapeutic approach (e.g., study of the blood brain barrier in order to achieve a better delivery of drugs).

Recently, different types of endothelial cells have been used in *in vitro* models to obtain organ-specific vascular models (39) and this is what we are also interested in.

### An Innovative Tool: The “Microvasculature-on-a-chip” Model

In order to test endothelial cells' behavior in a three dimensional dynamic model reproducing the influence of physiological flow and shear stress as an important part of “everyday life” of the endothelium, we developed and tested a “microvasculature-on-a-chip” microfluidic device (40).

**Figure 1:**
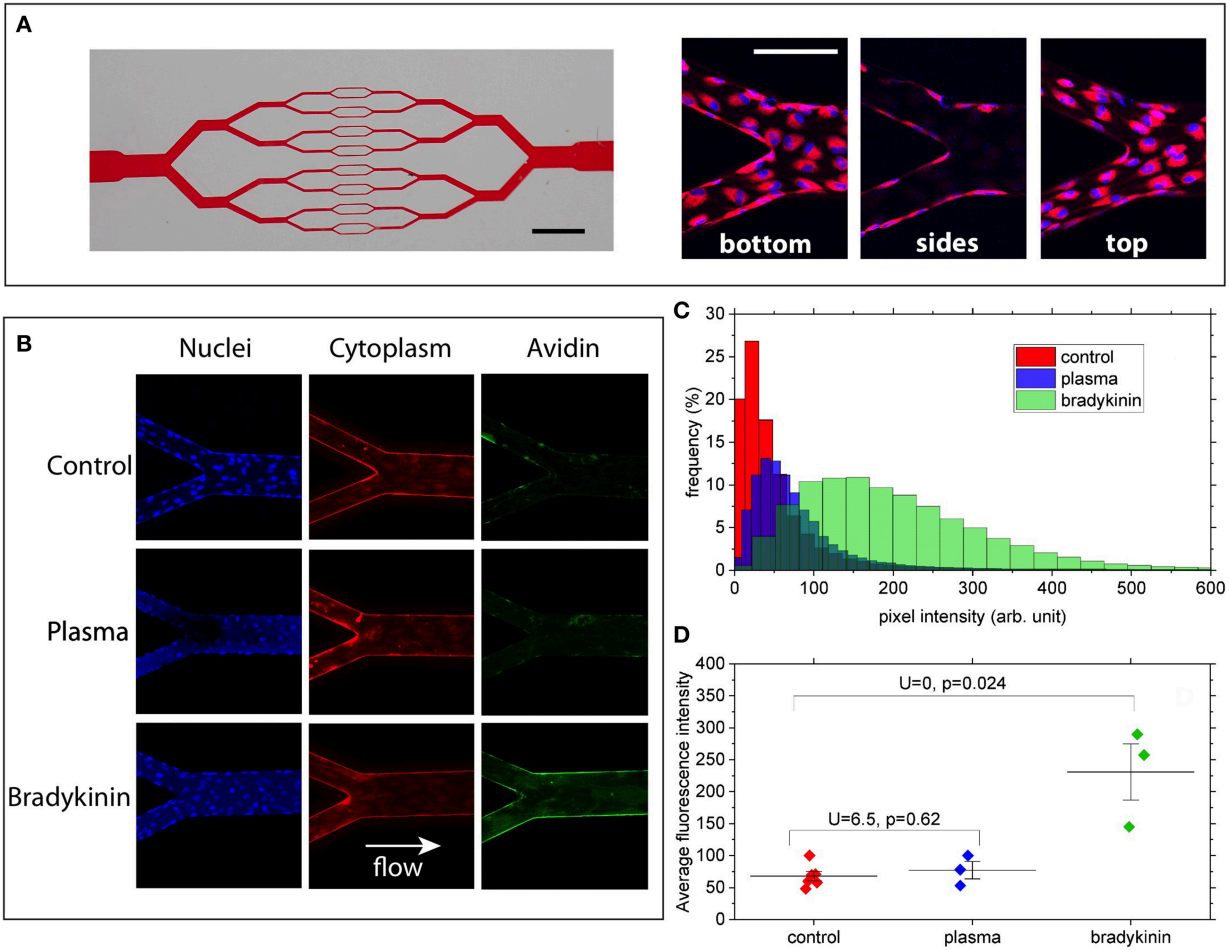
**(A)** Left: picture of the channel network illustrating the branching/converging geometry used (scale bar: 2 mm). Right: merged images showing cell nuclei (blue) and cytoplasm (red) at the bottom, on the lateral walls and at the top of the channels (scale bar: 100 μm). **(B)** Z-summed images obtained from 3D-stacks showing nuclei (blue), cytoplasm (red), and wall-bound avidin (green) for the 3 conditions: control, 50%-plasma, and bradykinin. Image size is 425 × 425 μm. Imaged regions correspond to a merging point between two 60 μm-wide channels and one 120 μm-wide. **(C)** FITC intensity histograms (relative frequency as a function of pixel intensity) computed for the 3 conditions from the images shown in **(A)**. **(D)** Average fluorescence intensity values measured for the various conditions over 3–6 different bifurcation areas of the networks. U and *P*-values correspond to two-samples Mann–Whitney *U*-tests showing that, at the 5% threshold, Bradykinin and Control data are different whereas Plasma and Control are not.

Briefly, the model consists of 30μm-high microchannels organized in a branching/converging network (Figure 1A). At each branching point the width of each channel is divided by two, reaching 30 × 30 μm (height × width, square section) in the middle part of the chip. Circuits were fabricated from PDMS and sealed with a glass coverslip at the bottom to allow high-resolution microscopy. Channel walls were coated with biotin-conjugated fibronectin (Cytoskeleton Inc, USA) as a matrix before seeding the circuit with Human Umbilical Vein Endothelial Cells (HUVECs, PromoCell, Germany), chosen as a commonly used human model to study endothelial functions and physiology. HUVECs were cultured within the networks, in the presence of a steady flow of culture medium, ensuring a physiologically relevant level of fluid shear stress at the wall of ~0.2 Pa. In the present condition HUVECs were able to adhere to all four walls of each channel and to form a confluent monolayer within a few days after seeding (Figure 1A).

After reaching confluence, HUVECs' cytoplasm was stained with CellTracker Red (Molecular Probes^TM^) and the cells were exposed for 15 min to a constant flow of one of the following media: (i) usual endothelial cells culture medium (PromoCell, Germany) as a control, (ii) plasma from healthy volunteer—blood was withdrawn in Sodium Citrate tubes, plasma was separated from cells by centrifugation and diluted 1:1 with medium for ECs culturing, (iii) bradykinin (Sigma, USA) diluted in endothelial cells culture medium at a concentration of 25 μM. FITC-conjugated avidin (Molecular Probes^TM^) was finally added to the perfusion solution (final concentration is 25 μg/ml for 5 min) before cells were fixed with 4% paraformaldehyde and their nuclei stained with Hoechst 33342 (Molecular Probes^TM^).

Laser Scanning Confocal Fluorescence Microscope (Zeiss, Germany) was used for cell imaging. 3D image stacks (425 × 425 × 35 μm in X, Y, and Z) of various parts of the microchannels were acquired with a 20x objective, with a sampling of 0.140 μm/pixel in the X/Y and 1.5 μm/pixel in Z direction. The acquired image stacks were processed and analyzed using the Fiji open-source platform (41). 3D stacks were first transformed into 2D images by projecting (summing) the pixel intensities along the Z direction, for the 3 detection channels corresponding to nuclei, cytoplasm and avidin (see Figure 1B).

Under our experimental conditions, high-affinity interactions between FITC-avidin and biotinylated-fibronectin can take place only when avidin molecules have crossed the endothelial layer. By monitoring the fluorescence level associated with the presence of FITC-avidin bound to the walls of the microchannels, we can therefore assess the permeability of the endothelial monolayer. This was done by computing intensity histograms from the Z-projected 2D images (Figure 1C). In order to quantitatively compare such histograms for the three different media to which HUVECs were exposed, we took care to acquire all stacks using the same imaging parameters, and to compute histograms from sections of the microchannels spanning the same surface (namely 49,000 μm^2^ here). Doing so, we observe that (Figure 1C):

- no major difference in histogram peak or width can be seen between the control and the 50%-plasma from healthy volunteer conditions,
- in the circuit exposed to 25 μM solution of bradykinin the histogram is clearly shifted to larger intensity values compared to that obtained for the control.

Moreover, such a trend is confirmed when comparing the average intensity values computed from histograms measured over 3–6 different regions of the microchannel networks (Figure 1D): we observe a significantly higher average value (I_Brad_ = 230 ± 76) for the bradykinin-treated channels as compared to the control circuit (I_Control_ = 68 ± 18), whereas no statistical difference is found between control and plasma (I_Plasma_ = 77 ± 23) conditions. Since the intensity of the fluorescent signal depends on the quantity of FITC-avidin bound to the surfaces of the channels, this result can be considered as a sign of increased endothelial permeability after its exposure to bradykinin.

This was further confirmed by negative controls (images not shown) where we have imaged channels coated with non-biotinylated fibronectin after exposure to FITC-avidin. When no cells were present in the channels (i.e., fibronectin freely accessible to avidin), we measure an average fluorescence intensity of 15 ± 8, barely different from the detection noise (I_noise_ = 10 ± 4), showing the very low level of non-specific binding of FITC-avidin to non-biotinylated fibronectin. With HUVECs cultured in such channels, we find an average intensity of 38 ± 6, with fluorescence being detected within the cytoplasm of cells but not at the channel walls, which suggests that a large fraction of the intensity detected in the control and plasma conditions above correspond to FITC-avidin internalized by the cells without reaching the underlying walls.

## Discussion

Under the term Paroxysmal Permeability Disorders, we would like to gather pathological conditions which probably share some common pathophysiological mechanisms and whose humanistic burden is remarkable not only because of possible lethality, but also because they can significantly affect patients' quality of life for long-term complication, social impact and anxiety related to fear of upcoming events. Increased knowledge and awareness of the pathophysiological mechanisms underlying these conditions has been the cornerstone for the development of new drugs in some cases (e.g., hereditary angioedema) and getting deeper insights is of pivotal importance in order to correctly treat these conditions and avoid acute and long-term complications.

In our study we propose to focus not only on soluble factors, mediators and receptors when investigating PPDs, but also on the endothelium itself as an active dynamic player with high impact on appropriate functioning of the vascular system. Our “microvasculature-on-a-chip” model can reproduce the size of real capillary beds and allows performing experiments in tightly controlled conditions: shear stress, specific fluid composition, varying humoral factors and mediators. Endothelial cells can be seeded into the systems, reaching a physiological confluent monolayer, expressing the main markers typical for endothelium including a glycocalyx lining the entire lumen of the channels (40). The microchannel networks are suitable to investigate normal and abnormal interactions between blood cells and vessel walls. In the next future endothelial cells from specific vessels' beds or from vessels with specific characteristics (e.g., microvascular endothelial cells) could be seeded into the system. It is also possible to conceive the elaboration of gene study assays, thanks to the possibility to collect cells from the device after challenge with different kinds of “stressors.” Even though our results are very preliminary, our study confirms that the proposed three dimensional dynamic model is able to detect changes in endothelial permeability. Since our previous work (40) demonstrated HUVECs' longevity in the channels under constant culture medium flow, restoration of the endothelial barrier after exposure to the agents increasing intercellular junctions permeability is expected. Therefore, our microfluidic device could be a promising powerful tool to get deeper insights into the mechanisms underlying PPDs.

It should be noted that our “microvasculature-on-a-chip” model still has some limitations. First of all, the stiffness of polydimethylsiloxane is different from that of the glass coverslip at the bottom of the circuit, so that the extravascular “microenvironment” is still not very close to physiology and changes of some endothelial functions have to be carefully interpreted between the sides of the circuit. However, new strategies involving the use of soft hydrogels and materials mimicking the interstitial matrix may allow investigations which are more consistent with the conditions *in vivo*, with a possibility to evaluate not only endothelial permeability to fluids, but also the transport of molecules and flowing cells. Further refinement of the fabrication and cell seeding procedures will allow higher automation and reproducibility which is of high importance when dealing with cells exposure to different kinds of fluids (e.g., RBC suspension with varying hematocrit levels), circulating factors and mediators. The design of a micro-chamber surrounding the microchannels network and filled with interstitial-like matrix will open the way to test in more details the response of the system to plasma (and eventually blood) from angioedema and ISCLS-patients (collected both during attacks and in intercritical periods) as well as cells' migration through the endothelial monolayer in both directions.

Despite the above-mentioned limitations, we believe that the presented system may be suitable to investigate new therapeutic approaches, including, for instance, the delivery of drugs via innovative nanotechnologies. When investigating new treatments for ISCLS, we would like to evaluate the effect of agonist and antagonist of Tie 2 as AKB-9778 and Angt2. We hypothesize that AKB-9778 might be a valuable strategy not only to treat or prevent diabetic retinopathy, but also to treat systemic conditions characterized by increased permeability, such as ISCLS. Finally, we speculate that angioedema and ISCLS should be regarded as “simple disease models” in the huge scenario of conditions characterized by hyperpermeability and that our “microvasculature-on-a-chip” model can be a powerful tool to advance in study and better understand alterations underlying this type of disorders.

Ethical approval was not required for this study in accordance with the local legislation and institutional requirements. Written informed consent was obtained from all participants.

## Ethics Statement

Involvement of human subjects was limited to blood withdrawals from healthy volunteers. All subjects gave written informed consent in accordance with the Declaration of Helsinki.

## Author Contributions

MW, DT, LiB, CM, and MC were responsible for the conceptualization of the study. DT, LiB, AD, and CM designed the microvasculature on a chip model. MW, IB-G, LaB collected blood samples from healthy volunteers; MW, DT, and MI developed the protocol to test endothelial permeability in the microfluidic device. MW, DT, and MI performed the experiments (from fabrication of the microchip and seeding of HUVECs to cell imaging with confocal fluorescence microscopy). MW, DT, and LiB were responsible for image processing and analysis. MW wrote the first draft of the manuscript. MW, DT, LiB, IB-G, AD, LaB, CM, and MC revised the original draft and helped in manuscript editing. CM and MC were in charge of supervision of all the steps of the study. CM and MC were responsible for project administration. CM, LiB, and MC provided the resources to develop the project.

### Conflict of Interest Statement

The authors declare that the research was conducted in the absence of any commercial or financial relationships that could be construed as a potential conflict of interest.
